# Influence of Affective Stimuli on Leg Power Output and Associated Neuromuscular Parameters during Repeated High Intensity Cycling Exercises

**DOI:** 10.1371/journal.pone.0136330

**Published:** 2015-08-25

**Authors:** Hamdi Jaafar, Majdi Rouis, Laure Coudrat, Thierry Gélat, Timothy David Noakes, Tarak Driss

**Affiliations:** 1 Laboratoire CeRSM (EA 2931), UFR STAPS, Université Paris Ouest Nanterre La Défense, Nanterre, France; 2 LCOMS, EPSAP, Emotion-action, UFR SciFA, Département STAPS-Metz, Université de Lorraine, Metz, France; 3 Department of Human Biology, University of Cape Town, Cape Town, South Africa; Victoria University, AUSTRALIA

## Abstract

The aim of this study was to examine the impact of emotional eliciting pictures on neuromuscular performance during repetitive supramaximal cycling exercises (RSE). In a randomized order, twelve male participants were asked to perform five 6-s cycle sprints (interspaced by 24 s of recovery) on a cycle ergometer in front of neutral, pleasant or unpleasant pictures. During each RSE, mean power output (MPO) and electromyographic activity [root mean square (RMS) and median frequency (MF)] of the vastus lateralis and vastus medialis muscles were analyzed. Neuromuscular efficiency (NME) was calculated as the ratio of MPO to RMS. Higher RMS (232.17 ± 1.17 *vs*. 201.90 ± 0.47 μV) and MF (68.56 ± 1.78 *vs*. 64.18 ± 2.17 Hz) were obtained in pleasant compared to unpleasant conditions (p < 0.05). This emotional effect persisted from the first to the last sprint. Higher MPO was obtained in pleasant than in unpleasant conditions (690.65 ± 38.23 *vs*. 656.73 ± 35.95 W, p < 0.05). However, this emotional effect on MPO was observed only for the two first sprints. NME decreased from the third sprint (p < 0.05), which indicated the occurrence of peripheral fatigue after the two first sprints. These results suggested that, compared with unpleasant pictures, pleasant ones increased the neuromuscular performance during RSE. Moreover, the disappearance of the beneficial effect of pleasant emotion on mechanical output from the third sprint appears to be due to peripheral fatigue.

## Introduction

It has been demonstrated that emotion impacts human performance in many situations such as during button press tasks [1], arm movement [2], wrist isometric contraction [3] or gait initiation [4]. For example, in the latter study, the initial whole body forward disequilibrium was higher in front of pleasant pictures, compared with unpleasant ones, suggesting that the initiation of forward gait was facilitated in a pleasant context. More recently, the beneficial effect of pleasant emotion on physical performance has been extended to a supra-maximal exercise, i.e., the repetition of five 6-s all-out cycling sprints separated each by a 24-s recovery period [5], hereafter referred as a kind of repetitive sprint exercise (RSE). The results of this previous study showed that mechanical output were higher in front of pleasant pictures, compared with unpleasant ones. In line with the central governor model (CGM) [6] and the proposal of Baron et al. [7], the results of the study of Coudrat et al. [5] demonstrated that pleasant and unpleasant emotional states induced by the perception of pleasant and unpleasant stimuli, respectively, have the potential to regulate exercise intensity. More precisely, the more pleasant the emotional state during exercise, as during the viewing of pleasant pictures, the greater the desire to maintain or to increase the exercise intensity [7]. However, this process appears to be transient since it was present only in the first two sprints and became similar between the pleasant and unpleasant conditions from the third to the fifth sprint [5].

Furthermore, several studies have shown that mechanical power output during RSE depends on central neural factors, i.e., the number of motor units recruited, as evidenced by the electromyographic (EMG) activity in the lower limb [8–10]. For example, changes in power output and EMG activity amplitude (root mean square, RMS) during a 10 × 6-s cycling RSE were strongly correlated [10]. In the CGM [6], EMG activity is thought to be centrally selected before or during exercise according to internal (e.g., from peripheral physiological systems) and external (e.g., environmental conditions) information [6, 11] in order to maximise motor output and maintain internal homeostasis [6]. This process reflects a planned cognitive strategy, named the pacing strategy, that has been highlighted during continuous exercise [11] and during RSE [8]. Therefore, it seems to be relevant to postulate that the higher power output observed in a pleasant context compared to an unpleasant one [5] could result from a higher central neural drive.

Moreover, it has been proposed that, when exercise intensity is high, as it is the case during RSE, internal information such as physiological feedback and sensation of exertion dominates the capacity of the nervous system to treat information, so that external stimuli become less influential [12]. If this theory is correct for RSE, it could be hypothesized that the disappearance of this emotional effect on power output from the third sprint observed in the study of Coudrat et al. [5] could result from the inability of the emotional effect to influence central neural drive as the exercise duration increases. However, to our knowledge, the impact of emotional factor on the central neural drive during RSE has not been studied.

On the other hand, the power output during RSE depends also on peripheral factors, i.e., the capacity of the skeletal muscle to generate force in response to central recruitment [9, 13–16]. As RSE measures the ability to produce and repeat maximal effort with incomplete recovery periods (i.e., < 30 s), fatigue could occur and resulting in a power decrement during repetitive sprinting exercises [17]. A reduction of the ratio between mechanical output and EMG activity, named the neuromuscular efficiency (NME), is considered a measure of the development of peripheral fatigue during exercise. This has been demonstrated during a 5 × 6-s cycling RSE, during which the decrement of EMG amplitude from the fourth sprint occurs in parallel to a reduction of NME in the fifth sprint [13]. Therefore, in the study of Coudrat et al. [5], it could be hypothesised that peripheral fatigue specifically induced by sprint repetitions could interfere with emotion, resulting in the disappearance of the beneficial effect of pleasant pictures on power output from the third to the fifth sprint. However, the study of Coudrat et al. [5] did not specifically address this hypothesis.

Therefore, the aim of the present study was twofold: i) To analyse the impact of emotion on EMG activity during RSE in order to confirm the Baron et al.’s proposal [7], according to which emotional factor can influence performance by acting on the extent of central motor drive. If this is the case, the EMG activity in the lower limb is expected to be higher in front of pleasant pictures, compared with unpleasant ones; ii) To verify whether the disappearance of the emotional effect on power output from the third to the fifth sprints during a 5 × 6-s cycling RSE results from either reduced central motor drive or the development of peripheral fatigue.

## Materials and Methods

### Participants

Twelve active and healthy males (28.58 ± 3.23 years, 82.41 ± 13.29 kg and 1.78 ± 0.05 m) gave written informed consent to participate in the study. Participants practiced recreational sporting activities for about 4–5 h a week but none of was a cyclist. The present data were collected as part of a larger study from which the preliminary results were presented in previous paper [5], in which the effects of emotion upon power output were examined. Participants were excluded from the study if they reported any of the following: i) a lower limb injury or muscle soreness, ii) any medical condition that would impair their ability to perform high intensity tasks. Participants received thorough explanations about the measurement procedures and the possible risks and benefits of participation in the study. The study was approved by the Institutional Review Board of the Nanterre University and was conducted according to the guidelines of the Declaration of Helsinki for human experimentation.

### Experimental design

Participants were familiarised with all experimental procedures and with exercising on the cycle ergometer but were blinded about the purpose of the study. Participants performed three experimental sessions (neutral, pleasant and unpleasant) in a randomized order, each consisted of a warm-up and a RSE cycle test [18].

#### Warm-up

The standardized warm-up consisted of 5 min of cycling at 80 W, followed by 3 min of rest and then a 10-s maximal sprint test on the cycle ergometer. Upon completion of the 10-s test, participants rested for 5 min before performing the RSE cycle test. Importantly, participants were required to gaze horizontally during the 10-s maximal sprint test. This instruction served as training to ensure that the participants maintained a horizontal gaze during the subsequent RSE cycle test, during which a projected picture had to be viewed for the duration of each 6-second sprint. The participants were informed that if their gaze fell below the horizontal during the RSE test, then the trial would be discarded.

#### RSE cycle test

Each RSE cycle test consisted of 5 × 6-s sprints separated each by 24-s of passive recovery on a cycle ergometer (Monark 894E, Stockholm, Sweden). The load was set at 60 g.kg^-1^ of body mass on the basis of previous results showing that a load between 50 and 75 g. kg^-1^ of body mass is suitable for the determination of maximal power in active adults [19]. Cadence data from an encoder placed on the cycle ergometer flywheel were recorded through the Monark Anaerobic Test Software. The mean power output (MPO), defined as the average power elicited throughout the 6-s of the sprint was calculated for each sprint.

Participants were seated on the cycle ergometre placed at a distance of 2.50 m in front of a white wall of the laboratory on which visual stimuli were presented. The laboratory ambient temperature was controlled at ~ 22°C.

At trial onset, adjustments to saddle height, foot position on pedals, and upper body position were made according to each subject’s satisfaction. Toe clips and heel straps were used to prevent the feet from slipping. The optimal riding position was maintained identical throughout the study. The start and end of each sprint was performed according to the onset and the extinction of a single visual stimulus, respectively. Participants were instructed to i) assume the ready position while awaiting the start visual signal; ii) pedal as soon as and as fast as possible after stimulus onset; iii) perform an all-out effort throughout the duration of the stimulus presentation and to look at the stimulus the entire time it was on the wall, i.e., do allow the gaze to deviate from the horizontal during the 6-s sprint test.

#### Stimuli presentation

As described in Coudrat et al. [5], SuperLab Pro v.2 was used to control the visual stimuli presented during each sprint which began with a 4-s preparatory period, characterized by the display of a fixation cross, followed by a 6-s visual stimulus (95 cm × 160 cm). During the familiarization session, the visual stimulus was a white square. During the three test sessions, it was a neutral, pleasant or unpleasant image. Thus, 15 images were selected from the International Affective Picture System (IAPS) according to their normative ratings for male participants [20]. These images represented neutral people for the neutral session, sports images for the pleasant session and mutilation for the unpleasant session.

IAPS image numbers were: i) neutral, 2102, 2191, 2383, 2513, 2570; ii) pleasant, 8060, 8090, 8200, 8210, 8260; iii) unpleasant, 3015, 3051, 3062, 3068, 3100. All participants were naïve to the IAPS images.

Immediately following the completion of all sessions, a computerized 9-point version of the self-assessment manikin (SAM) [20] was used to obtain subjective ratings of valence and arousal of all images viewed during the sessions. In this system, ratings of valence are indicated by five graphical representations of facial expressions ranging from a severe frown (most unpleasant) to a broad smile (most pleasant). For the arousal, the manikin varies from a state of low to high agitation. Participants may select any of the five figures, or boxes in between.

#### EMG activity

During each sprint, the surface EMG of the vastus lateralis and the vastus medialis of the right leg were continuously recorded using a wireless EMG unit (ZeroWire Aurion, Italy). The EMG signals were collected by a pair of adhesive bipolar surface electrodes (Universal Ag/AgCl electrodes, FIAB, Florence, Italy). Recording electrodes were fixed at a constant inter-electrode space of 2 cm over the muscle belly placed longitudinally to the underlying muscle fibre direction according to the recommendations of SENIAM [21], i.e., at 2/3 of distance between the anterior superior iliac spin and the lateral aspect of the patella for the vastus lateralis and at approximately 20% of the distance between the medial gap of the knee joint and the anterior superior iliac spine for the vastus medialis. In order to ensure reliable electrode replacement throughout the experimental period, the electrode sites placement were marked with indelible felt-tip pen [22]. Before the beginning of every EMG testing, the overlying skin was shaved, lightly abraded and cleaned with an alcohol pad to remove dirt and oil. The EMG transmission units were fastened to the participant’ limb with medical adhesive tape and wrapped in an elastic bandage to avoid artefacts from lower limb movements. The electromyograms were amplified (common mode rejection ratio of 92 dB, input impedance below 2000 Ω, gain = 1000) at a sampling capture rate of 1000 Hz and filtered to a bandwidth between 20 and 450 Hz.

Information about muscle activity was considered for both amplitude and frequency. Muscle activity amplitude was quantified as the root mean square (RMS) of the signal between the onset and offset of activation of every burst. The onset and offset of activation of all EMG bursts during each 6-s sprint were detected using a constant electrical threshold of 0.2 mV [23]. The RMS value of each burst was averaged within each 6-s sprint. Muscle activity frequency was quantified as the median power frequency (MF) of each EMG activity muscle. This was performed using AcqKnowledge 4.2 Software (Biopac Systems Inc., Santa Barbara, CA). A decrease of the MF indicates a decrease in the muscle fibres conduction velocity [24, 25].

Global RMS and MF indices were calculated as the averaged of the RMS and MF values of the two muscle groups, respectively. In addition, neuromuscular efficiency (NME) was calculated for each sprint as the ratio between MPO and global RMS activity. NME was used as an indicator of the peripheral muscle contractility [26]. A NME reduction indicates the occurrence of reduced skeletal muscle contractility.

#### Perceptual responses to exercise

For each 6-s sprint, participants were asked to rate their subjective perceived exertion (RPE) using the 6–20 point Borg scale [27]. The end of each 6-s sprint corresponded to the offset of a picture. At this time, the RPE scale appeared on the wall and participants had to report one number from 6 (very very light) to 20 (very very hard) [27].

### Statistical analysis

Statistical analyses were processed using Statistica 7.1 Software for Windows version (StatSoft, Maisons-Alfort, France). Prior to any statistical analyses, each variable was tested for normality using the Shapiro-Wilk test. Average cadence, Global RMS, MF, MPO, NME and RPE were examined using a two-way analysis of variance (ANOVA) with repeated measures [pictures (3) × sprints (5)]. The difference between the ratings of self-reported valence and arousal of each image category was analysed using one-way repeated-measures ANOVA (image categories: neutral, pleasant and unpleasant). *Post hoc* analyses were conducted using the Bonferroni test. All significance thresholds were set at p < 0.05. Effect sizes were calculated as partial eta-squared (η^2^) to estimate the meaningfulness of significant findings.

## Results

### Pictures ratings and psychological indices

According to the IAPS model, valence of each image category was chosen by the experimenter as significantly different from each other (p < 0.05). Sports images (6.9) were rated as more pleasant than neutral people images (5.1), which were rated as more pleasant than mutilation pictures (2.2). However, while the arousal of sports and mutilation pictures were not significantly different (p = 0.480), neutral people images were rated as less arousing (p < 0.001) than both sport and mutilation images (neutral people: 3.2, sport: 6.1, mutilation: 5.8).

The one-way ANOVA performed on the ratings of self-reported valence (SAM procedure) revealed a main effect of image category (F_2,22_ = 156.45, p < 0.001, η^2^ = 0.93). *Post hoc* testing showed that valence for pleasant image (6.3 ± 0.4) was significantly higher (p = 0.01) than neutral images (5.3 ± 0.2); the latter being significantly higher (p < 0.001) than unpleasant ones (1.2 ± 0.1).

The one-way ANOVA performed on the ratings of self-reported arousal also revealed a main effect of image category (F_2,22_ = 93.81, p < 0.001, η^2^ = 0.89).In contrast with normative arousal ratings (IAPS), post hoc test demonstrated that pleasant images (4.5 ± 0.5) were rated as less arousing (p < 0.001) than unpleasant ones (7.8 ± 0.3). Neutral images (2.3 ± 0.4) were rated as less arousing (p < 0.001) than pleasant and unpleasant images.

**Fig 1:**
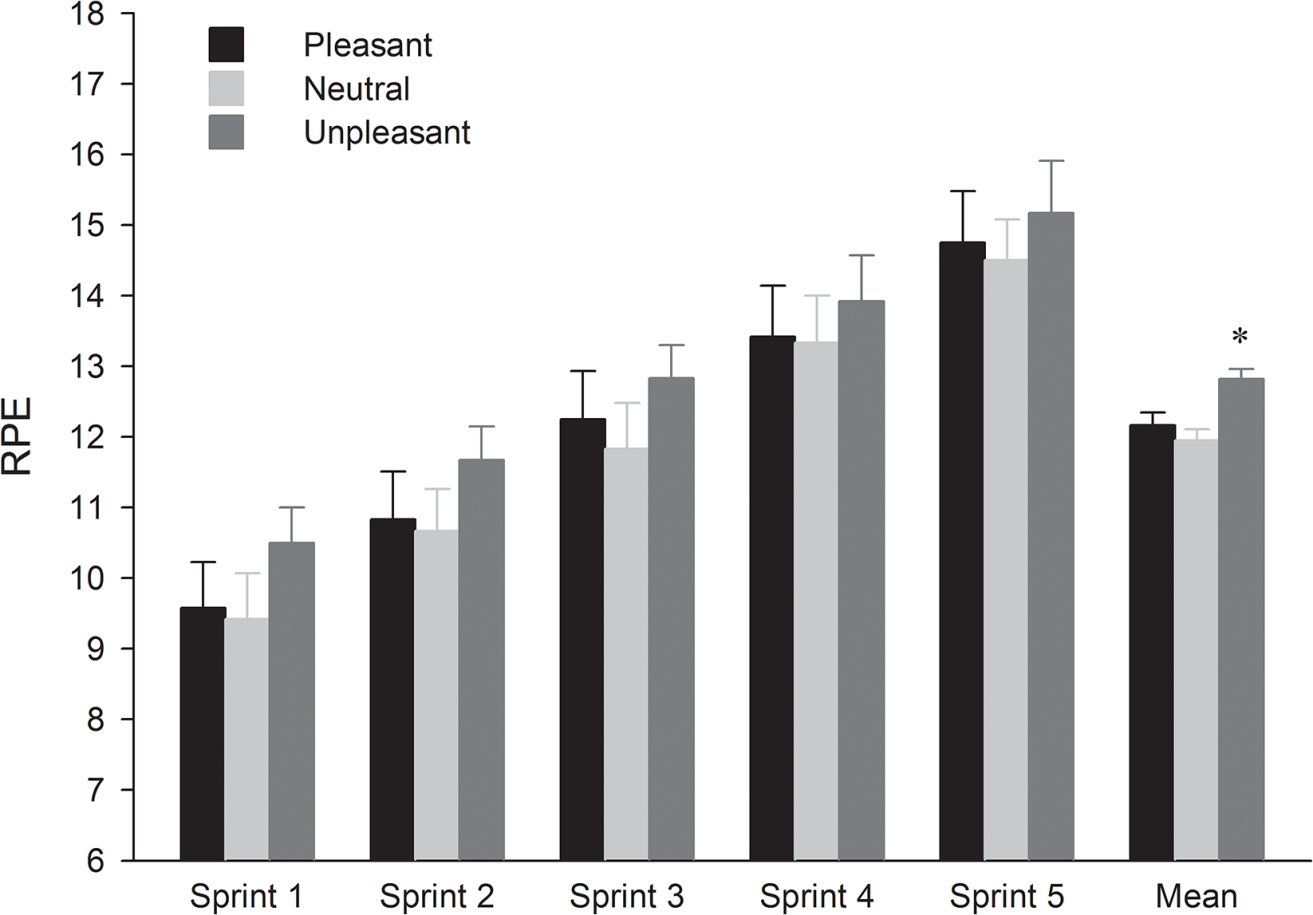
Mean (and standard error) across the pictures category and sprints for ratings of perceived exertion (RPE). * Significantly higher compared to neutral condition (p < 0.05).

Regarding the rating of perceived exertion (RPE) (Fig 1), the two way ANOVA showed a main effect of sprint (F_4,44_ = 37.08, p < 0.001, η^2^ = 0.77). *Post hoc* testing revealed that RPE increased from sprint 3 (p < 0.001), regardless of the image category. Moreover, the two way ANOVA demonstrated a main effect of picture category (F_2,22_ = 4.59, p = 0.021, η^2^ = 0.29). Exposure to unpleasant pictures led to significantly higher RPE as compared with neutral pictures. However, no difference (p > 0.05) was found between the neutral and pleasant sessions and between the pleasant and unpleasant ones. Moreover, no interaction image × sprint was found (p > 0.05) on this variable.

### Cadence

The results of average cadence are reported in Table 1. A significant main effect of picture category was observed on cadence (F_2,22_ = 20.37, p < 0.001, η^2^ = 0.65). *Post hoc* testing showed that cadence was significantly higher in neutral and pleasant conditions compared to unpleasant ones but there was no difference between neutral and pleasant pictures. A significant main effect of sprint exercise duration was observed on cadence (F_4,44_ = 38.99, p < 0.001, η^2^ = 0.78). Post hoc test showed that cadence decreased significantly from the second sprint. A significant picture category × sprint interaction effect was observed (F_8,88_ = 3.92, p = 0.001, η^2^ = 0.26). *Post hoc* test showed that cadence was higher in the pleasant and neutral sessions than in the unpleasant one in the two first sprints and was higher in the pleasant session than in the unpleasant one in the final sprint (Table 1).

**Table 1:** Average cadence (rpm).

		Sprint 1	Sprint 2	Sprint 3	Sprint 4	Sprint 5
**Pleasant**	*Mean*	*151.25	*144.33	137.58	134.17	*130.25
	*SD*	12.86	11.38	12.32	10.9	9.19
**Neutral**	*Mean*	*147.75	*141.92	137.67	132.5	128.75
	*SD*	11.66	12.65	11.64	11.29	11.04
**Unpleasant**	*Mean*	139.33	135.58	133.75	129.92	125.25
	*SD*	14.85	12.75	10.31	9.65	11.47

* Significantly higher compared to the unpleasant condition (p < 0.05).

### Power output

**Fig 2:**
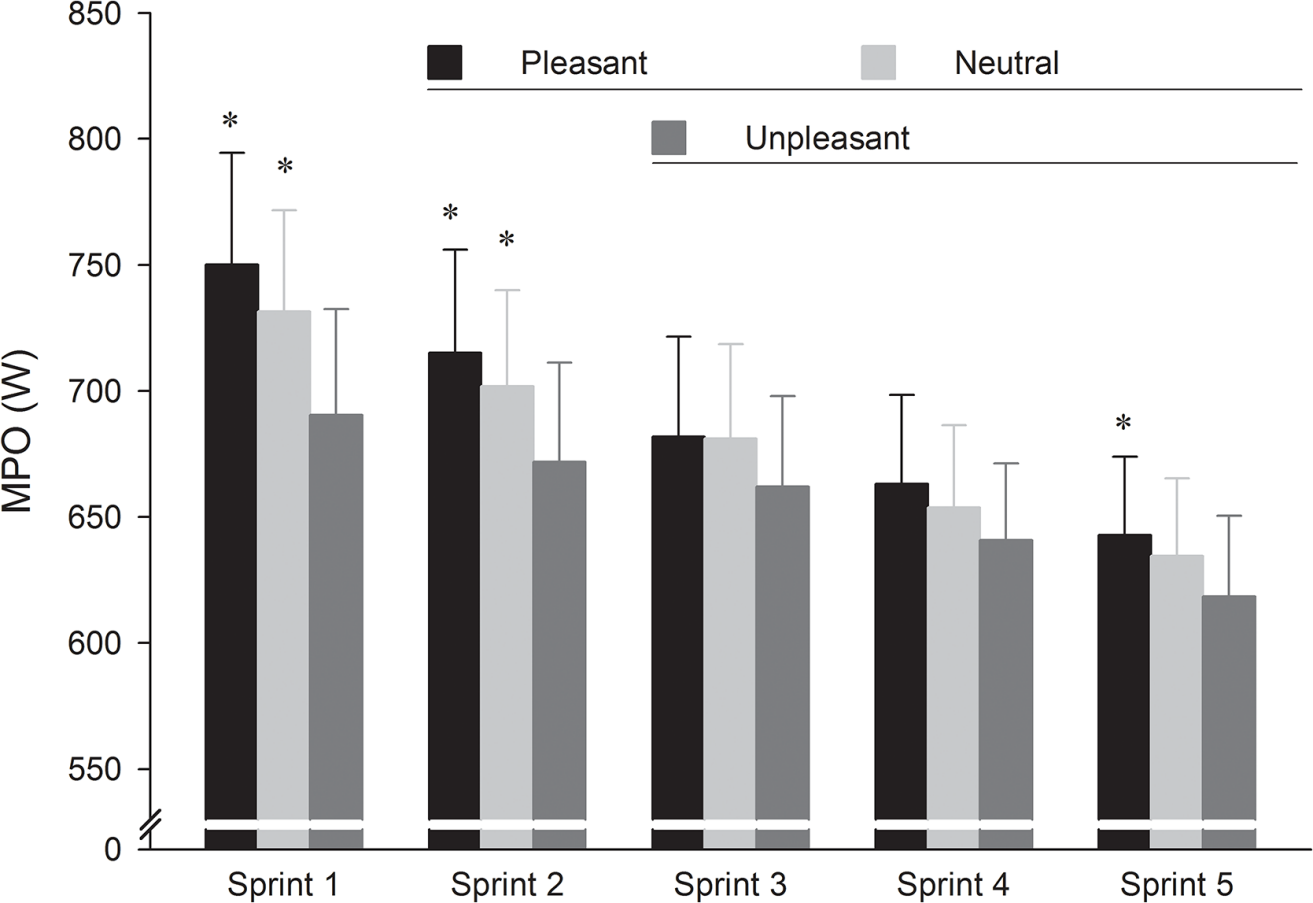
Mean (and standard error) across the pictures category and sprints for mean power output (MPO). When compared to the first sprint, MPO decreased significantly (horizontal line) from the second sprint in the neutral (grey bars) and pleasant (white bars) sessions and from the third sprint in the unpleasant (black bars) session. * Significantly higher compared to the unpleasant condition (p < 0.05).

The two-way ANOVA performed on MPO showed a significant main effect of picture category (F_2,22_ = 20.61, p < 0.001, η^2^ = 0.65). *Post hoc* testing revealed that MPO was significantly higher in neutral and pleasant pictures compared to unpleasant ones but there was no difference between neutral and pleasant pictures (p > 0.05). The two-way ANOVA demonstrated also a significant effect of the sprint exercise duration (F_4,44_ = 39.02, p < 0.001, η^2^ = 0.78). *Post hoc* test showed that, when compared with the first sprint, MPO decreased significantly from the second sprint. Moreover, the picture × sprint interaction was also significant (F_8,88_ = 3.93, p < 0.001, η^2^ = 0.26). *Post hoc* test showed that MPO was higher in the pleasant and neutral sessions than in the unpleasant one in the two first sprints and was higher in the pleasant session than in the unpleasant one in the final sprint (Fig 2). Moreover, when compared with the first sprint, there was a significant (p < 0.05) decrease in MPO in the neutral (4.1%) and pleasant (4.7%) sessions during the second sprint and in the unpleasant (4.1%) session during the third sprint (Fig 2). Over the five sprints, MPO decreased by 14.3%, 13.2% and 10.4% in the pleasant, neutral and unpleasant sessions, respectively, compared to the value for the first sprint.

### EMG activity

**Fig 3:**
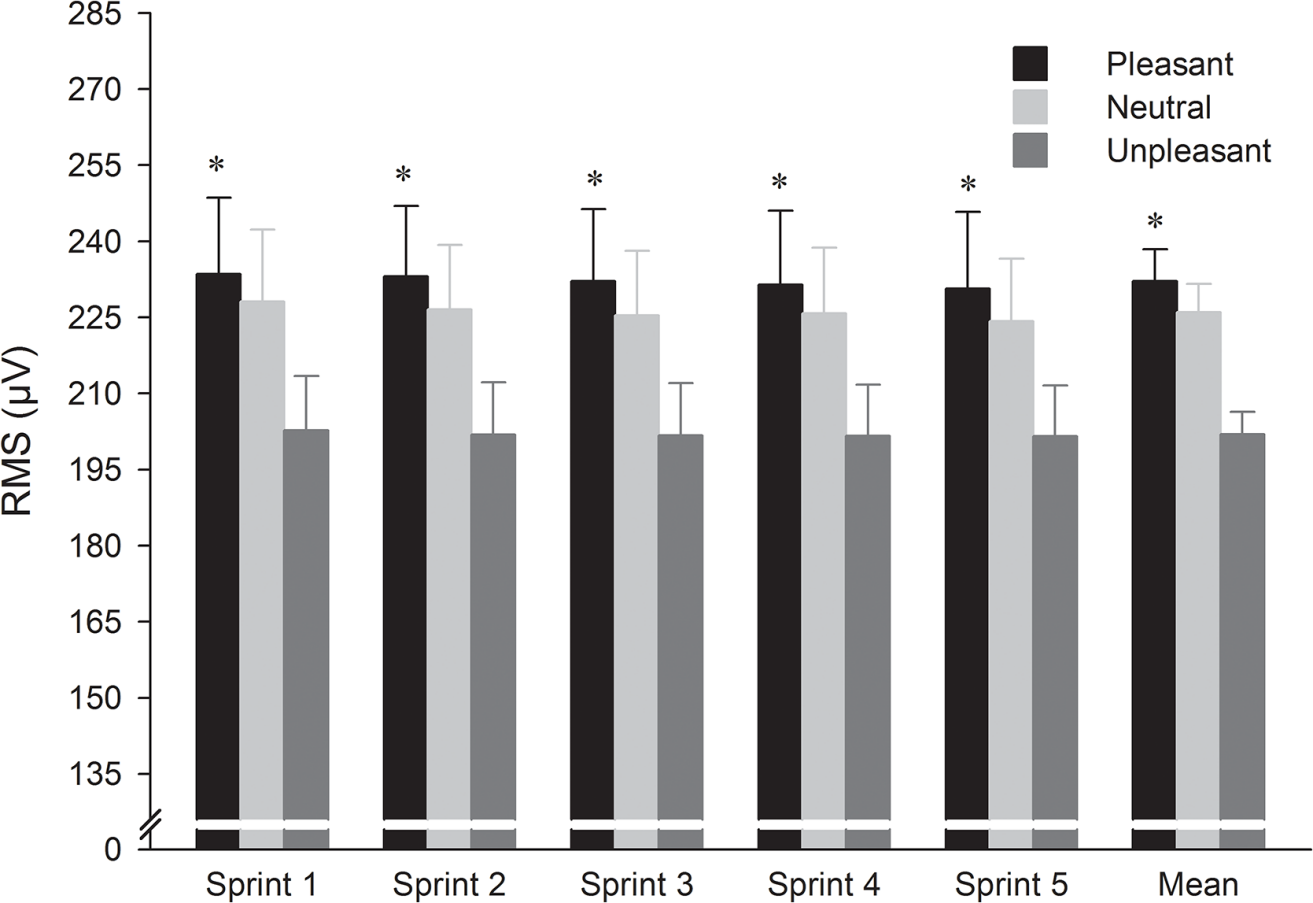
Mean (and standard error) across the pictures category and sprints for root mean square (RMS). * Significantly higher in the pleasant compared to the unpleasant condition (p < 0.05).

The two-way ANOVA performed on the global RMS activity showed only a significant effect of picture category (F_2,22_ = 5.26, p < 0.05, η^2^ = 0.32). The global RMS activity was higher when viewing pleasant compared with unpleasant pictures but there was no difference between pleasant and neutral (p > 0.05) or unpleasant and neutral (p = 0.069). No significant main effect of sprint number and interaction picture category × sprint number were found on this parameter (Fig 3).

**Fig 4:**
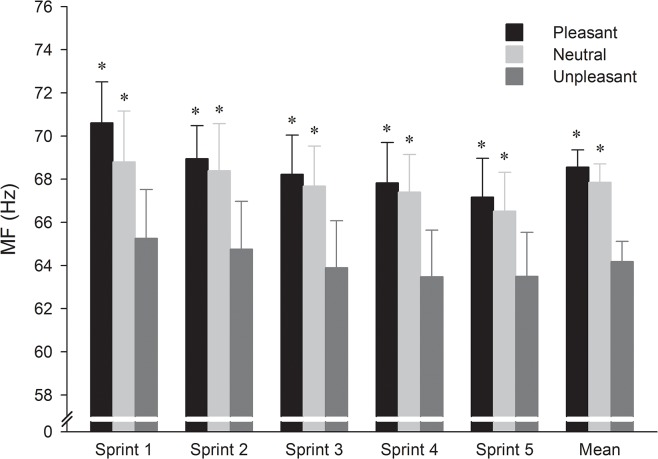
Mean (and standard error) across the pictures category and sprints for median frequency (MF). * Significantly higher compared to the unpleasant condition (p < 0.05).

The analysis of global MF (Fig 4) showed significant effects of picture type (F_2,22_ = 6.72, p < 0.01, η^2^ = 0.38) and sprint number (F_4,44_ = 11.16, p < 0.001, η^2^ = 0.50). *Post hoc* testing revealed that MF was significantly higher in pleasant and neutral conditions, as compared with the unpleasant condition but not when pleasant and neutral conditions were compared (p > 0.05). In addition, MF decreased significantly from the third sprint compared with the first sprint. No significant interaction picture category × sprint number was observed.

### Neuromuscular efficiency

**Fig 5:**
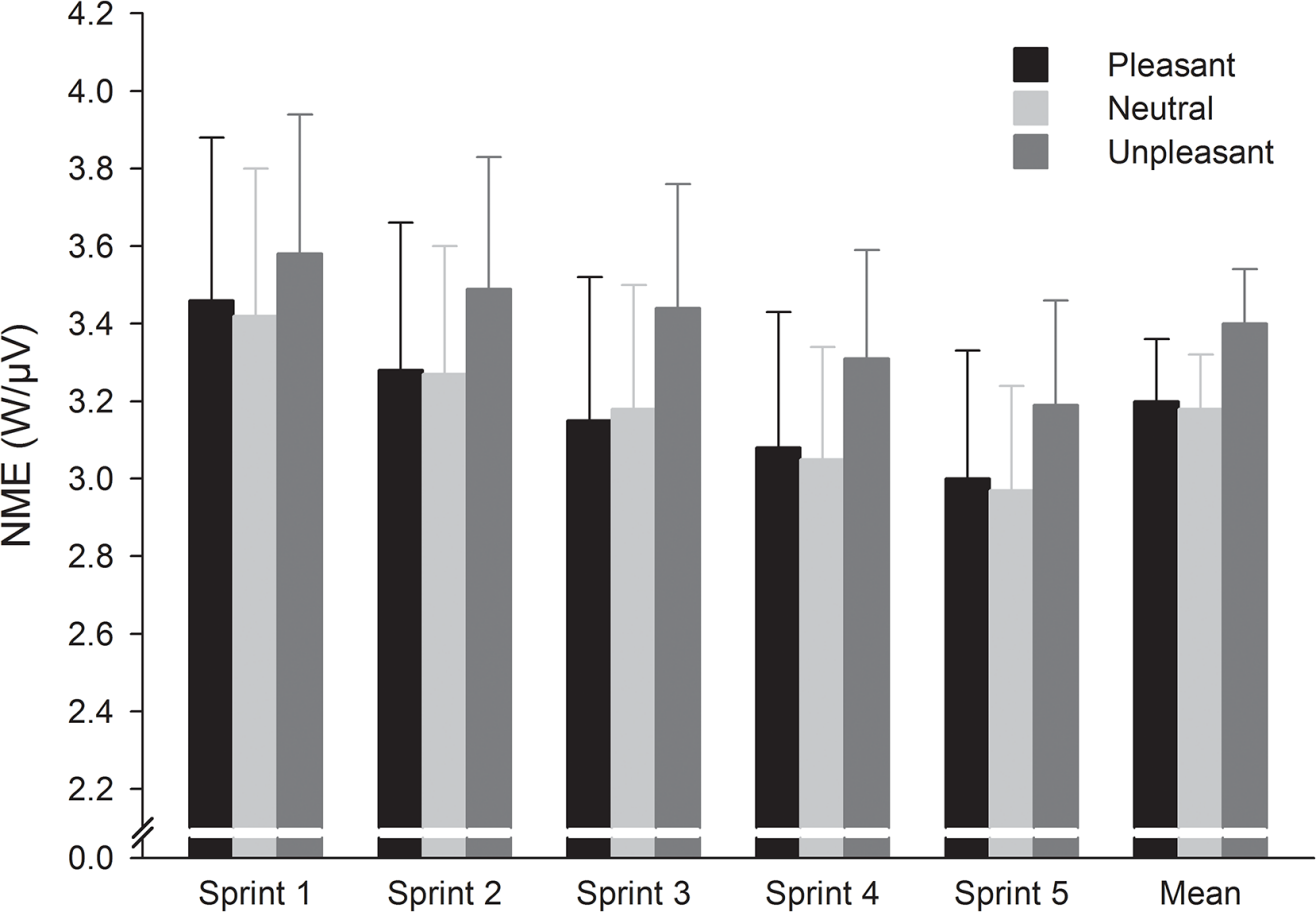
Mean (and standard error) across the pictures category and sprints for neuromuscular efficiency (NME).

The analysis of NME demonstrated only a significant main effect of sprint number (F_4,44_ = 13.35, p < 0.001, η^2^ = 0.55) (Fig 5). The results showed that, when compared with the first sprint, NME decreased significantly (p < 0.05) from the third sprint. No significant main effect of picture category and interaction picture category × sprint number were found.

## Discussion

The first aim of the present study was to confirm the finding of Baron et al. [7], according to which emotion centrally impact performance during RSE by altering the number of motor units that are recruited. The second aim of the present study was to determine whether the disappearance of the emotional effect on power output from the third to the fifth sprint during a 5 × 6-s cycling RSE [5] could result from an impairment of the central motor command or was due to the development of impaired skeletal muscle function (i.e., peripheral fatigue) with successive sprints.

In line with the proposal of the CGM [6] and the findings of Baron et al. [7], the results of the present study showed that emotion centrally impacts neuromuscular performance during a 5 × 6-s cycling RSE. Indeed, the higher mechanical output observed during the first two sprints in the pleasant condition occurred in parallel with higher amplitude of the EMG activity, compared with the unpleasant condition. Moreover, the global MF was higher in the pleasant than in the unpleasant condition. Combined, these results establish that the number of motor units recruited and the muscle fibres conduction velocity were both increased in the pleasant, than in the unpleasant context.

Moreover, while the impact of emotion on mechanical output disappeared from the third sprint, its effect on the central neural drive persisted until the final sprint. This occurred despite the appearance of increase level of physical discomfort and peripheral fatigue, as evidenced by an increase in RPE and a decrease in NME from the third sprint. This is a crucial point as it has been proposed that, when exercise intensity is high, as it is the case in the present study, physiological feedback dominates the capacity of the nervous system to process information, so that external information (i.e., emotional pictures) would no longer influence the outcome [12]. Nevertheless, RPE during the final sprint of the present study did not exceed 15, even though participants knew that the trial was essentially over. Therefore, it could be argued that the low level of perceived exertion even at the end of the exercise bout in the present study allowed for the central nervous system to continue processing emotional information perceived from the external environment.

The sustained effect of emotion on neural drive observed in the present study was consistent with previous studies. Indeed, a long lasting effect of emotion on behaviour during button press task as the number of trials increased [1]. In that study, when participants were required to press a button in response to a visual detection task, reaction time was faster when the task followed the passive viewing of a pleasant or neutral picture than after viewing an unpleasant image [1]. This effect persisted through the repetition of 24 trials, each following a picture of the same valence [1]. As in this previous study, the sustained effect of emotion on neural drive during RSE observed in the present study could be due to the induction of an altered emotional state, which likely resulted from the activation of appetitive and defensive motivational system in the pleasant and unpleasant contexts, respectively [28].

The present study also found that the higher neuromuscular performance in the pleasant compared to the unpleasant condition was not associated with a higher RPE. Instead, RPE was similar in both conditions. In the CGM [6], RPE is thought to be one manifestation of alterations in neural drive, so that an increase in EMG activity, as it was observed in the pleasant condition of the present study, would have been expected to increase the sensory perception of fatigue. However, several studies have found that perceived exertion also has an affective component and depends on psychological factors such as affect, mood or associative thinking [11, 29]. For example, Baden et al. [29] found that the increase in unpleasant affect during a treadmill running bout was associated with the increase in RPE. Thus, as suggested by the study of Coudrat et al. [5], the finding in this study that participants produced a higher neuromuscular performance in the pleasant than in the unpleasant condition may be interpreted as the effect of a regulatory process to experience the exercise as equally pleasant so that the RPE did not decrease even though the exercise intensity increased.

In contrast with the results obtained for neural drive, results obtained on mechanical output revealed a transient effect of emotion. During the two first sprints, pleasant emotion allowed increases in both neural drive and associated mechanical output, compared with the effects of unpleasant emotions. However, from the third sprint, while neural drive was higher in the pleasant context, than in the unpleasant one, mean power output was similar in both conditions. These results suggested the disappearance of the emotional effect on mechanical output from the third to the fifth sprint. Importantly, the neural drive was maintained in all sprints repetitions, regardless of emotion, suggesting that the central motor command was not impaired with trial repetition. However, the results of the present study showed a decrease in NME from the third sprint, suggesting the occurrence of impaired skeletal muscle contractility (i.e., peripheral fatigue) after two first sprints. Therefore, it could be assumed that peripheral factors acting on the contractile presses negated the beneficial effects of pleasant emotion on mechanical output from the third to the fifth sprints. This result was consistent with previous studies [13, 26], according to which mechanical output during a 5 × 6-s cycling RSE is highly depended on peripheral alterations in skeletal muscle function.

## Conclusions

The results of the present study showed that emotional pictures impacted neuromuscular performance during a 5 × 6-s cycling RSE as a result central (neuronal) effects. Compared with unpleasant pictures, pleasant pictures increased both the number of motor units recruited and the muscle fibres conduction velocity. Moreover, while the beneficial effect of pleasant emotions on these central mechanisms persisted throughout the five sprints, the effect on mechanical output was limited to the first 2 sprints only. These results occurred in parallel with the decreased of NME from the third sprint, suggesting the occurrence of peripheral fatigue with sprints repetition (i.e., reduction in skeletal muscle contractile function). Considered together, these results have interesting implications for training programs that focus on muscle power enhancement. First, our results suggest that pleasant emotional pictures could be used as external stimuli to increase neuromuscular performance. Second, it appears that a delay in the occurrence of peripheral fatigue might be an option that would preserve the beneficial effects of pleasant emotion on mechanical power output during repeated sprint exercise.
